# Considerations for Adapting Pre-existing Mechanistic Quantitative Systems Pharmacology Models for New Research Contexts

**DOI:** 10.3389/fphar.2019.00416

**Published:** 2019-04-18

**Authors:** Michael Weis, Rebecca Baillie, Christina Friedrich

**Affiliations:** Rosa & Co. LLC, San Carlos, CA, United States

**Keywords:** quantitative systems pharmacology, modeling and simulation, virtual patient, pharmacometrics, *in silico* modeling, biomedical research, drug discovery and development

## Introduction

Adapting existing models for new uses is an attractive strategy for quantitative systems pharmacology research due to the presumed savings on cost, time, and resources vs. creating new models from scratch. However, the process can present significant technical and scientific challenges and should be undertaken with appropriate expectations.

## Body

As mechanistic modeling becomes more widely adopted in drug development, models are increasingly available for possible reuse. Publications and websites such as the BioModels Database (www.biomodels.net) and Drug Disease Model Resources (www.ddmore.eu) offer hundreds of mechanistic representations of biological pathways. With these available resources, drug development professionals must decide whether to adapt one of these existing models for new research or create a model from scratch. Potential time, cost, and resource savings, along with the ability to leverage others' efforts and expertise, are motivating reasons to use existing mechanistic models or their components. But can published (or otherwise pre-existing) models be utilized for new QSP research?

Some prominent examples from literature suggest that reuse of existing mechanistic models is feasible. For example, computational approaches to modeling blood pressure regulation were pioneered by Guyton et al. in the early 1970s (Guyton et al., 1972) and have been frequently updated and adapted (Hallow and Gebremichael, 2017). Similarly, a 2013 review (Ajmera et al., 2013) traced the genealogy of 100+ models of glucose homeostasis and diabetes developed over the past 50+ years, and an early osteoclast/osteoblast model (Komarova et al., 2003) is cited in a later model incorporating additional pathways (Lemaire et al., 2004), which is cited in later models of osteoporosis (e.g., Peterson and Riggs, 2012), and multiple myeloma-induced bone disease (e.g., Ji et al., 2014). In the authors' own organization, previously developed model components and prior existing models frequently inform the models developed for new research contexts.

Several recent publications have provided guidance on best practices for documenting and publishing models to support future use of the models. For example, Cucurull-Sanchez et al. (2019) present a “minimum set of recommendations that can enhance the quality, reproducibility and further applicability of QSP models” and suggestions for “how to document QSP models when published, framing a checklist of minimum requirements”. This perspective aims to address the complementary angle—how to evaluate existing models and consider repurposing them for new research purposes and contexts.

Adapting pre-existing models requires a high level of care. Models must be relevant, correct, and credible to ensure impact. Regardless of whether a model is new or pre-existing, modeling decisions should be made with the research context in mind. Components of the research context include: the key research question(s) or decision(s) to be made, available data and knowledge, time and resource constraints, and the cross-functional project team and management team members (Friedrich, 2016). Even the best models will not be of use if the decision makers do not have confidence in the results. Criteria for assessing if an existing model is fit for a research context should include technical considerations, model scope, biological uncertainty and variability, and model testing (Table 1). Each of these criteria is considered below.

**Table 1:** Criteria and considerations for adapting models.

**Criteria**	**Considerations**
Scope	• Does the model:
	° Represent appropriate biology?
	° Include necessary biological components and processes?
	° Include appropriate level of biological detail (especially for your target areas)?
	° Represent the appropriate timeframe (e.g., minutes vs. years)?
	° Represent the phenotype (e.g., therapeutic area, severity) of interest?
	• Is the size and complexity appropriate to the time and resources you can apply?
	• Is the biology represented appropriately?
	• Is the embedded biological knowledge current?
	• Is the original research context clear?
	• Are assumptions clearly stated?
	• Are assumptions appropriate for the new research context?
	• Are data and parameter sources appropriate for the new research context?
Uncertainty	• Does the publication identify key knowledge gaps and associated assumptions?
	• Does the publication evaluate the impact of key uncertainties via sensitivity analysis or “what if” scenario testing?
	• Does the publication include multiple Virtual Patients (VPs) to explore biological uncertainty that is relevant to the new research context?
Variability	• Does the publication identify known pathway variability?
	• Does the publication evaluate the impact of pathway variability via sensitivity analysis or “what if” scenario testing?
	• Does the publication comment on clinical variability?
	• Are multiple relevant VPs included?
	• If VPs are included, how do they differ from each other mechanistically?
	• If VPs are included, what clinical phenotype and response to therapy do they represent?
Testing	• Qualitative Testing:
	° Were relevant experts consulted to assess if model results looked reasonable?
	° Were relevant sources of information for qualitative testing identified and used, e.g., clinical data from related therapeutic areas, or relevant non-clinical data?
	° Were what-if experiments performed to assess model behavior?
	° Are subsystem behavior tests described, with appropriate data references?
	• Quantitative Testing:
	° Were relevant clinical data for the drug of interest used for testing?
	° Were relevant clinical data for drugs in the same therapeutic area used for testing?
	° Were multiple disparate types of model perturbations tested and compared to relevant data?
	° Did the model perform adequately, given the new research context?
	° Does the model include relevant clinical outcome measures and/or biomarkers?
	° Is it clear how the outcome measures were derived from the represented biology?
	° Were population-level outcomes reproduced with appropriate range and distribution of outcomes?

### Technical Considerations

Reproducibility is a fundamental requirement for reuse. However, in most cases, published models have technical issues that must be resolved before the model runs. For example, over 90% of curated models at one online repository could not be reproduced (Sauro, 2016). The most common challenges are related to typographical errors, incomplete specification of the model or simulation protocols, inconsistencies between the text and supplied model code, and the unavailability of model code or software environments. Even if the model source code is provided, it often is inadequately documented, or lacks the full specification of simulation protocols required to reproduce published results (Kirouac et al., 2019). These challenges have hindered the wide-scale use of online model databases, and the regular reuse of published models.

To overcome such issues, recent publications suggest adopting best practices for ensuring model reproducibility and reuse (Cucurull-Sanchez et al., 2019; Kirouac et al., 2019). Technical standards, such as Systems Biology Markup Language (SBML) and Simulation Experiment Description Markup Language (SED-ML), are being developed to address some of these issues. However, resolving technical issues is only the first step toward ensuring that a model is appropriate for use. Even if a model runs and reproduces published results, care must be taken to evaluate the model's suitability and the appropriate questions must be asked. Why was the model built? Under what conditions is the model valid? How was the model qualified? What biological uncertainties and variabilities were identified during the model development process? Has the impact of these uncertainties or variabilities on model outcomes been evaluated?

### Model Scope Evaluation

Having clarity on the research context is essential before building or adapting a model because scope and modeling decisions must be made with the research context in mind. For example, a pathway may be modeled in more detail than it otherwise would be if a target on the pathway is to be modulated, or certain mechanisms relevant for long-term regulation may need to be added to extend a model's temporal scope. If a pre-existing model is to be used, researchers should assess what the original research context was and what adaptations are needed to ensure that the model is appropriate for the new research context.

Even if a model appears to be well-suited to be used in a new research context, it is advisable to conduct a formal process of answering and documenting the questions in Table 1 to ensure understanding and stakeholder buy-in. Stakeholder understanding of, and confidence in, the model is essential for ensuring impact of the modeling project.

As an example, consider adapting a model for use in diabetes research, which has a long history of mathematical modeling (Ajmera et al., 2013). The BioModels Database lists 64 diabetes models; and more are available on PubMed. The decision to select a published model might depend on desired outcome measures, study protocols, duration of study, intervention, and acceptance of the model by the broader research community. If the diabetes model is to be used, for example, for a multi-year prediction, then it should account for biological processes that change on the weeks-to-months timescale (e.g., insulin resistance), whereas these processes may be excluded (i.e., assumed constant) if the goal is to assess the acute impact of a novel target. Clearly defining the new research context and scope can make a seemingly overwhelming task of selecting an appropriate model more manageable. In the authors' experience, even if a suitable model is identified, it is expected that some components will need to be added to adapt it to the new research context.

### Biological Uncertainty and Variability

There is uncertainty in biology, so mechanistic models must make assumptions about uncertain pathways. Uncertainties may be qualitative (e.g., is there a compensatory feedback loop that may limit the effect of inhibiting a target?) or quantitative (e.g., what is the exact strength of the feedback effect?).

Uncertainty arises from lack of data; reproducibility of data; methods for generating, analyzing, and reporting data; as well as the scientist's or modeler's interpretation of the data (Fourier et al., 2015; McShane, 2017). For example, a recent publication summarized the significant variability and its specific sources in reports of circulating chemokine and immune cell levels in inflammatory bowel disease (IBD) patients (Rogers et al., 2018), including assay differences, choice of cell markers, summary analysis methods (e.g., grouping subjects as responders vs. non-responders), and diversity in the resulting summary statistics reported (e.g., mean or median, standard deviation or interquartile ranges, etc.).

Mechanistic models facilitate the evaluation of the relative importance of these uncertainties and the possible risks they may pose. Once the material uncertainties have been identified, additional modeling steps can help evaluate the impact of alternative hypotheses, e.g., by using Virtual Patients (VPs) (Friedrich and Paterson, 2004; Rullmann et al., 2005; Shoda et al., 2010; Kirouac, 2018). Briefly, a VP is one complete parameterization of a model that represents one feasible set of biological hypotheses of disease pathophysiology and is consistent with relevant test data. Using multiple VPs can substantially reduce risk in pharmaceutical decision making.

The determination of which uncertainty matters most depends on the research context. Most publications include only limited discussion of uncertainties, and this may not be focused on the most relevant biological uncertainties for the new research context. For example, if a novel target is being evaluated, the uncertainty around that specific target warrants a closer look. Even if a publication includes an analysis of the impacts of uncertainties via sensitivity analysis or VPs, the results may differ for the new research context. Different parts of the system may be instrumental in driving different outcomes (e.g., different pathways are key drivers for fasting plasma glucose vs. hepatic glucose production), and sensitivities may shift for different patient types, so sensitivity analysis results may identify different drivers (and corresponding material uncertainties) depending on the focus of the research. Assessing and documenting uncertainty helps to provide context for future creation of VPs.

Variability is another crucial consideration in pharmaceutical research. It is often critical to assess patient heterogeneity because patients differ in their pathophysiology (mechanistic pathway variability), clinical presentation, and/or response to therapy (Chung and Adcock, 2013; Chung, 2016). This variability is separate from variability introduced by data measurement and analysis. Patients are inherently variable due to natural causes (e.g., genetics, age, comorbidities, environment) (Dibbs et al., 1999). VPs can be used to capture and explore the aspects of patient variability relevant to the new research context. New VPs can be added to a model if the existing set of VPs (if any) do not explore all aspects of mechanistic variability thought to be relevant to the new research context.

### Model Testing

Testing is a critical step in adapting an existing model. Existing models are often under-tested for the new research context. Furthermore, many publications fail to fully describe the testing procedures or results. Models are constrained by a variety of data types including: physical laws and constraints, health and disease physiology, target and drug mechanisms, preclinical pharmacology, marketed therapies, and clinical trials for the investigational compound(s). Testing, therefore, should be appropriate for the type of available data such as qualitative vs. quantitative, subsystem vs. whole-system behavior, healthy vs. disease physiology, and individual vs. population-level, and described clearly. The modeling team and key stakeholders should decide which tests are most relevant to ensure that the model is fit for its intended purpose.

It is important to consider whether the original data used to construct and qualify the model is still appropriate for the new research context. For example, IBD studies often group Crohn's disease and ulcerative colitis into a single category. An IBD model may therefore not be appropriate for reuse, without considering whether the data used for model qualification is representative of the intended study population. Both disorders may involve similar components (e.g., immune and epithelial cells), and the pathology may be represented by similar model structures. However, the two disorders are significantly different. Using an IBD dataset to test a model might be appropriate under select assumptions, but inappropriate if the new research focus is a single disease. The original model parameters which assumed similar cellularity, mediator concentrations, and/or rate constants may not be appropriate with the new research focus.

## Conclusions

There is more to a model than equations and parameters. To have meaningful impact, model scope must be relevant to the new research context (fit-for-purpose), relevant exploration of uncertainties and variability should be considered as part of the process of adapting the model, the model must be tested against relevant data to ensure it behaves appropriately, and clinical and management team members should be involved to ensure credibility. If an existing model does not meet all relevant criteria for the new research context, additional modeling and qualification activities should be expected. While adapting a pre-existing model for a new research context is often feasible, modeling teams should do so with appropriate expectations.

## Author Contributions

All authors listed have made a substantial, direct and intellectual contribution to the work, and approved it for publication.

### Conflict of Interest Statement

MW, RB, and CF are employees of Rosa and Co. LLC.
